# Associations between prepartum urine pH and periparturient blood calcium concentrations in multiparous Holstein cows

**DOI:** 10.3389/fvets.2025.1649751

**Published:** 2025-08-20

**Authors:** Jesse P. Goff, Pedro Melendez, Julian A. Bartolome, Thomas R. Overton, Brittany M. Leno, Geneva Graef, James K. Drackley, Kristen M. Glosson, Xiangfei Zhang, Stephen J. LeBlanc, Rita Couto-Serrenho, José E. P. Santos, Camilo Lopera, Roney Zimpel, Rachael M. Rodney, Ian J. Lean

**Affiliations:** ^1^Biomedical Sciences, College of Veterinary Medicine, Iowa State University, Ames, IA, United States; ^2^Veterinary Clinical Sciences, Jockey Club College of Veterinary Medicine and Life Sciences, City University of Hong Kong, Hong Kong, Hong Kong SAR, China; ^3^Facultad de Ciencias Veterinarias, Universidad Nacional de La Pampa, General Pico, Argentina; ^4^Department of Animal Science, Cornell University, Ithaca, NY, United States; ^5^Department of Animal Science, University of Illinois, Urbana, IL, United States; ^6^Department of Population Medicine, University of Guelph, Guelph, ON, Canada; ^7^Department of Animal Sciences, University of Florida, Gainesville, FL, United States; ^8^Faculty of Veterinary Science, University of Sydney, Camden, NSW, Australia

**Keywords:** hypocalcemia, urine pH, DCAD, acidification, milk fever

## Abstract

**Introduction:**

Metabolic alkalosis induced by prepartum diet cations impairs Ca homeostasis in the periparturient cow. Adding anions to prepartum diets reduces blood pH improving periparturient Ca homeostasis. Urine pH generally reflects blood pH and is practical to measure on farm. The degree to which urine should be acidified to increase periparturient blood Ca concentration is not well defined.

**Materials and methods:**

Prepartum urine pH and periparturient blood Ca concentrations determined in 660 multiparous Holstein cows from 9 studies were analyzed. Least Square Means of the lowest blood Ca concentration (Ca nadir) observed in cows within 6 urine pH categories (≤ 5.75, 5.76 to 6.25, 6.26 to 6.75, 6.76 to 7.25, 7.26 to 7.75, and ≥ 7.76) were determined. Data were analyzed across all 660 cows and then by parity.

**Results:**

Across all cows, the Ca nadir was lowest and the incidence of cows with Ca nadir < 2.00 mM, indicative of subclinical hypocalcemia (SCH), was greatest when urine pH was ≥ 7.76. Mean Ca nadir increased in all cows with urine pH < 7.75. There was no significant difference in Ca nadir or % of cows with SCH when prepartum urine pH was < 7.75. This was also the case for 2nd and 3rd parity cows. However, in ≥ 4th parity cows, those with urine pH between 6.26 and 6.75 had significantly higher Ca nadir than cows with urine pH above 7.25 or below 5.75. Calcium nadir and blood Ca concentrations at 2 days in milk (DIM) were highly correlated (r = + 0.58), and blood Ca concentrations at 2 and 4 DIM were moderately correlated (r = + 0.43). Blood Ca concentration at 4 DIM was weakly associated with Ca nadir (r = + 0.30).

**Discussion:**

These analyses support acidification of cows to achieve prepartum urine pH below 7.75 to increase blood Ca nadir for 2nd and 3rd parity cows. For ≥ 4th parity cows, the highest blood Ca nadir was observed with urine pH below 7.25 and above 5.75. For ≥ 4th parity cows, urine pH below 5.75 was associated with significantly lower blood Ca nadir.

## 1 Introduction

Clinical hypocalcemia is common in dairy cows starting their second or greater lactation in the hours before and especially in the first 2 days after calving. The high Ca content of colostrum (2.2 to 2.4 g/L) and transition milk causes a rapid, large demand for Ca that challenges the ability of many cows to maintain normal blood Ca concentration [> 2.0 m*M* on the day of calving and > 2.20 m*M* by 4 days in milk (DIM)]. Cows rely on parathyroid hormone to initiate mechanisms such as bone osteocytic osteolysis and osteoclastic Ca resorption to return low blood Ca concentrations to normal concentrations. Parathyroid hormone also stimulates renal conversion of 25-hydroxyvitamin D to the hormone 1,25-dihydroxyvitamin D, which stimulates transcellular intestinal Ca absorption, greatly increasing the efficiency of dietary Ca absorption (1). In some cows, the degree of hypocalcemia is extensive enough to cause nerve tetany and muscle paresis resulting in recumbency and the clinical disorder known as milk fever. Cows developing milk fever have poorly functioning immune cells, reduced DMI, and greater risk of ketosis, mastitis, metritis, retained placenta and displaced abomasum (2). Subclinical hypocalcemia (SCH) affects many more cows than does milk fever (3, 4). Hypocalcemia, by various definitions, is associated with impaired immune cell function (5, 6), greater risk of subsequent disease or culling, reduced reproductive performance, and, conditional on timing or persistence, with lesser milk yield (7, 8).

Alkalogenic diets, which are typically high in K and other cations impair Ca homeostasis mechanisms of the periparturient cow, which can result in acute periparturient hypocalcemia (9, 10). Late gestation cows generally are fed diets high in forages and therefore high in non-metabolizable cations, particularly K. The diet difference in the milliequivalents (mEq) of absorbable cations and anions (DCAD) is a major determinant of blood pH (11). Typical diets, without addition of acidogenic products, are alkalogenic, and if fed to the cow prior to calving induce a metabolic alkalosis. Alkaline blood pH impairs the response of bone and kidney cells to parathyroid hormone stimulation (12–14). Studies also indicate alkalosis of the blood and extracellular fluids suppresses parathyroid hormone secretion (15), whereas metabolic acidosis increases parathyroid hormone secretion (16). Older cows suffer more severe hypocalcemia than younger cows around the time of parturition (3). Participation of bone in calcium homeostasis is greatly reduced in older cows compared to 1st parity cows. Evidence suggests there is a reduction in conversion of 25-hydroxyvitamin D to 1,25-dihydroxyvitamin D as well, and that the number of receptors for 1,25-dihydroxyvitamin D also declines with age (1).

While determination of blood pH in the prepartum cow is possible on farm it is currently expensive. In general, urine pH provides a reasonably accurate surrogate of blood pH, especially between urine pH of 5.9 to 8 (17). Urine pH has been used to determine whether a prepartum “anionic” diet has acidified the cows sufficiently to reduce the risk of hypocalcemia (18, 19). The optimal urine pH associated with hypocalcemia prevention remains controversial. Published studies often consist of 2 prepartum diets, one with a positive DCAD and the other with a negative DCAD. In these studies, cows fed the diet with the negative DCAD generally had an acidic mean urine pH and improved periparturient blood Ca concentration compared to the cows fed the positive DCAD diet that had an alkaline mean urine pH. As a result, linear correlations between mean urine pH values and the incidence of milk fever or the mean periparturient blood Ca concentrations have caused some meta-analyses to conclude that the lower the urine pH, the higher the blood Ca concentration of the periparturient cow (20). A meta-analysis by Santos et al. (7) examined mean blood total Ca concentration in 42 studies involving 1,652 multiparous cows. Most of those cows were Holstein. For that meta-analysis, means from each study were weighted based on precision of the data, defined as the inverse of the variance squared. They reported that mean blood total Ca concentration on the day of calving increased linearly as DCAD decreased. They also observed urine pH decreased as DCAD decreased but the relationship was quadratic, reaching a plateau above 8.0 when DCAD exceeded + 200 mEq/kg DM. Pre-partum DCAD of the studies utilized in that meta- analysis ranged from + 1,094 to – 246 mEq/kg DM.

Melendez et al. (21) analyzed the relationship between prepartum urinary pH and plasma total Ca concentration at 1 DIM in individual cows. The relationship was quadratic: cows with urine pH < 6 or > 7 had lower blood Ca concentrations than cows with urine pH between 6 and 7. Our objective was to expand upon this observation and describe the association between prepartum urine pH and postpartum blood Ca concentrations in a larger number of cows. We pooled data from 660 multiparous cows in 9 published studies to test the hypothesis that the lower the prepartum urine pH, the higher the blood Ca concentration after calving.

## 2 Materials and methods

### 2.1 Description of studies contributing data for analysis

Data from 9 published studies on prepartum DCAD effects on Ca status of multiparous periparturient Holstein cows were used in this analysis (Table 1). Criteria for inclusion were that individual cow urine pH was measured before calving and individual cow blood total Ca concentration was measured at least once during the first 24 h after parturition. The studies used various commercial anion supplements, alone, or mixed with anionic salts such as Ca or ammonium chloride or Mg sulfate to reduce the DCAD. Seven of the nine studies had positive DCAD “control” diets with cows having alkaline urine. In addition to DCAD, dietary Ca, Mg, and P content differed across the studies. Though these factors also influence both acid–base balance and the degree of hypocalcemia experienced by the cow (7), dietary Ca, Mg and P content effects were not considered in this analysis. Relevant reported diet information is summarized in Table 1. The data were received from the corresponding authors of each paper and were collated by J. P. Goff. No data were removed from the data sets provided. Within the individual studies some cows were excluded as described in each publication. The reasons for exclusion of cows assigned to the various dietary treatments in these studies generally involved abortion or still birth, error in breeding date, cows that calved too soon after placement onto the treatment diet, physical injury pre-calving, or dystocia. Cows carrying twins were excluded in some studies but not all.

**Table 1:** Description of diets used, remarks on experimental conditions, and number of multiparous cows in the 9 studies providing individual cow prepartum urine pH and postpartum blood Ca concentrations.

Study and reference #	# Cows	Study diets	DCAD	Diet Ca %	Diet *P* %	Diet Mg%	Remarks about study
Lopera et al. (2018) (28)	113	A B C	+109 −66 −176	0.67 0.64 0.62	0.33 0.33 0.33	0.44 0.47 0.48	Diets fed for 21 or 42 d before calving. No effect of time fed on blood Ca concentrations.
Goff et al. (2020) (22)	24	A B	+196 −9	0.66 0.65	0.25 0.24	0.47 0.46	Did not collect blood at 2 DIM
Rodney et al. (2018) (40)	51	A B	+138 −127	0.61 0.54	0.32 0.33	0.38 0.39	Vitamin D source was another fixed effect (calcidiol vs. cholecalciferol), but had no effect on hypocalcemia
Goff and Koszewski (2018) (24)	61	A B C	+167 −13 −17	0.46 0.46 0.72	0.25 0.25 0.24	0.58 0.58 0.44	Adding limestone increased urine pH
Couto Serrenho et al. (2021) (8)	67	A B	+105* −108*	1.2 1.28	0.35 0.28	0.45 0.43	Pen level trial on four farms. *Diet averages across farms.
Melendez et al. (2021) (21)	60	A B	−109* −129*	0.44 1.02	0.30 0.40	0.44 0.48	Cows from 2 pasture-based farms. *Noted wide variation in anion and pasture intake among cows
Leno et al. (2017) (29)	106	A B C	+183 +59 −74	1.54 1.57 1.57	0.44 0.43 0.41	0.47 0.48 0.50	
Graef et al. (2021) (25)	98	A B C D	−84 −20 −118 −29	1.5 1.5 0.74 0.67	0.34 0.35 0,33 0.34	0.50 0.49 0.49 0.47	Provided no data on day 4 blood Calcium concentration
Glosson et al (2020) (23)	80	A B C	+60 −240 −241	0.40 0.44 1.97	0.42 0.43 0.44	0.43 0.44 0.43	Adding limestone did not affect urine pH

DCAD (mEq/kg DM) = (mEq Na + mEq K) – (mEq Cl + mEq S).

In some cows the blood sample representing 2 DIM or 4 DIM also represented the time point of the Ca nadir and the data of these cows were not used in determining the correlation between Ca nadir and blood Ca concentrations at 2 DIM or 4 DIM. Six percent of the 660 cows experienced their Ca nadir on day 3 or after, and in all these cows the Ca nadir was above 2.00 mM. No correction was made for these cows and the Ca nadir of these cows is used in all calculations involving Ca nadir. Studies varied in the number of cows and the parity of the cows included. For example, Goff et al. (22), used only cows entering their third or greater parity. Glosson et al. (23) used 63 cows entering their 2nd parity and 13 cows entering their 3rd parity. Goff and Koszewski (24) provided no data on blood Ca concentration at 2 DIM. Graef et al. (25) provided no data on blood Ca concentration at 4 DIM. No cows in the studies were treated with oral Ca supplements. Several cows across the studies developed clinical milk fever. A blood sample was obtained prior to intravenous Ca administration, and this was deemed the Ca nadir for that cow. Blood total Ca concentration was determined on serum or plasma by inductively coupled plasma mass spectrometry (ICP-MS), atomic absorption spectrophotometry, or photometry using the Arsenazo III chemistry method. Four studies sampled cows 1 or 2 times during the first 24 h after calving. Five studies obtained 3 blood samples in the first 24 h after calving. The lowest blood Ca concentration observed in each cow is referred to as the blood Ca concentration nadir.

Urine pH was determined using calibrated pH meters on samples obtained by manual stimulation of micturition. Studies varied in the frequency of urine pH determination before calving. All cows had urine pH recorded at least once within the final 15 d of gestation, with most cows having urine pH determined during the final wk. of gestation. The urine pH recorded closest to the calving date was used in this analysis. In 16 cows the last urine pH was collected the day before calving. This value was not used as dry matter intake and thus diet anion consumed often decreases just prior to calving. In these cases, the urine pH value determined on the next closest day to the time of calving was used. Urine pH categories were formed to examine the effect on blood Ca nadir. We determined the mean blood Ca concentration nadir of cows within the 6 urine pH categories: ≤ 5.75, 5.76 to 6.25, 6.26 to 6.75, 6.76 to 7.25, 7.26 to 7.75, and ≥ 7.76. In the studies that fed the cows a diet with no supplemental anions added, urine pH of all cows was above 7.75. This formed our highest urine pH grouping. In an earlier model, two high urine pH categories were formed – those cows with urine pH above 8.25 and those with urine pH between 7.75 and 8.25, and two lower urine pH categories were formed (below 5.25 and 5.25–5.75) for a total of 8 groups. This reduced the number of cows at the extreme ends of urine pH that we are presenting in Tables 2, 3 and did not substantially alter the interpretation of the data. The strength of this data set is keeping a relatively high number of cows in each urine pH category so in the model presented those extreme urine pH groupings were combined.

**Table 2:** Parity distribution, number of cows diagnosed with clinical hypocalcemia within each parity, milking frequency/day, frequency of urine pH determination per week, within each of the 9 studies that contributed prepartum urine pH and post-partum blood Ca concentrations.

Study and reference #	# Cows of each Parity (# cows with clinical hypocalcemia)							MilkFreq^a^	Urine pHFreq^b^
Parity	2	3	4	5	6	7	8		
Lopera et al. (2018) (28)	48 (2)	41 (1)	15 (1)	6 (0)	2 (0)	1 (0)	0	2	2
Goff et al. (2020) (22)	0	15 (1)	5 (2)	4 (1)	0	0	0	2	2
Rodney et al. (2018) (40)	19 (4)	21 (3)	7 (1)	3 (1)	1 (0)	0	0	2	2
Goff and Koszewski (2018) (24)	19 (0)	9 (0)	14 (2)	10 (2)	4 (0)	2 (0)	1 (0)	2	2
Couto Serrenho et al. (2021) (8)	26 (0)	24 (0)	6 (0)	4 (0)	4 (2)	3 (0)	0 (0)	2 Farms −2 2 Farms-AMS^c^	1
Melendez et al. (2021) (21)	8 (0)	18 (2)	8 (1)	12 (0)	8 (0)	2 (0)	4 (2)	2	1
Leno et al. (2017) (29)	59 (2)	31 (0)	14 (2)	7 (2)	0	1 (0)	0	3	3
Graef et al. (2022) (25)	55 (0)	19 (0)	13 (0)	3 (0)	2 (0)			3	3
Glosson et al (2020) (23)	63 (5)	17 (0)	0	0	0	0	0	3	3

^a^Number of times /day cows were milked.

^b^ Number of times /week urine pH was determined during the study.

^c^ AMS= automatic milking system (robot).

**Table 3:** Effects, across all multiparous cows, of urine pH category and parity on blood Ca concentration nadir, % of cows with blood Ca concentration nadir below 2.00 mM, % cows classified as being sub-clinically hypocalcemic at 2 DIM, and % of cows classified as being dyscalcemic with blood Ca below 2.2 mM at 4 DIM.

Urine pH category	Ca nadir	% cows with Ca nadir < 2.00 mM	% SCH at 2 DIM	% Dyscalcemia at 4 DIM
≥ 7.76	1.66 ± 0.03^b^ *N* = 201	87 ± 3^a^ (175/201)	43 ± 4^a^ (71/166)	35 ± 6^a^ (65/185)
7.26 to 7.75	1.84 ± 0.05^a^ *N* = 58	75 ± 7^b^ (44/58)	29 ± 7^ab^ (13/45)	41 ± 7^a^ (20/48)
6.76 to 7.25	1.88 ± 0.05^a^ *N* = 49	57 ± 9^bc^ (28/49)	23 ± 7^b^ (10/42)	25 ± 7^a^ (10/38)
6.26 to 6.75	1.94 ± 0.04^a^ *N* = 67	58 ± 9^b^ (39/67)	26 ± 6^b^ (16/60)	45 ± 9^a^ (24/53)
5.76 to 6.25	1.91 ± 0.04^a^ *N* = 105	57 ± 8^c^ (60/105)	31 ± 5^ab^ (29/93)	34 ± 8^a^ (27/78)
≤ 5.75	1.88 ± 0.04^a^ *N* = 180	58 ± 7^c^ (104/180)	28 ± 4^b^ (49/176)	37 ± 6^a^ (49/132)
Parity	Ca nadir	% cows with Ca nadir < 2.00 mM	% SCH at 2 DIM	% Dyscalcemia at 4 DIM
2	1.99 ± 0.03^a^ *N* = 298	41 ± 6^c^ (122/298)	20 ± 3^b^ (54/272)	27 ± 5^b^ (61/227)
3	1.87 ± 0.04^b^ *N* = 195	63 ± 6^b^ (123/195)	27 ± 4^b^ (48/176)	41 ± 6^a^ (68/165)
≥ 4	1.70 ± 0.04^c^ *N* = 167	87 ± 4^a^ (145/167)	45 ± 0.4^a^ (60/134)	41 ± 6^a^ (58/142)

Numbers in parentheses are frequency of the disorder, SCH or Dyscalcemia, per the number of cows in each cell. Least Square Means + Standard errors. *N* = number of cows in each Table cell. ^a, b, c^Within column, different superscripts denote differences across urine pH category and across parity in the continuous LS means of blood Ca concentration nadir blood Ca using the method of Tukey–Kramer to correct for multiple comparisons (*p* < 0.05). ^a, b, c^Within column, different superscripts denote differences across urine pH category and across parity in the categorical LS means for % cows with blood Ca concentration nadir <2.00 mM, % cows with SCH at 2 DIM, and % cows with dyscalcemia at 4 DIM, using the method of Tukey–Kramer to correct for multiple comparisons (*p* < 0.05).

Since the risk of hypocalcemia increases with each parturition (26), the data were analyzed with all 660 multiparous cows, and further stratified into cows entering their 2nd (*n* = 298), 3rd (*n* = 195), or ≥ 4th parity (*n* = 167).

### 2.2 Statistical analysis

The association between prepartum urine pH and blood Ca concentration nadir using single measurements of each as continuous data was examined using mixed-effects models with the MIXED procedure of SAS (SAS/STAT version 9.4; SAS Institute Inc., Cary, NC) with the fixed effects of the linear covariate of urine pH, the quadratic covariate of urine pH, parity group (2 vs. 3 vs. ≥ 4), and the interactions between the linear covariate of urine pH and parity group, and the quadratic covariate of urine pH and parity group, and the random effect of experimental study. If the interactions with parity group resulted in *p* > 0.10, then they were removed from the final statistical model. Concurrently with removal of an interaction, the corrected Akaike’s information criterion (AICc) was evaluated and removal was performed if the AICc was reduced. The Kenward-Roger method was used to calculate the approximate denominator degrees of freedom for the *F* test in the statistical models.

When data were available, correlations between urine pH and blood Ca concentration on 2 or 4 DIM were also determined in separate models. Associations between blood Ca concentration nadir and blood Ca concentration at 2 and 4 DIM were also assessed. Only one blood sample was obtained at 2 DIM for some studies, between 25 and 48 h after calving. In other studies, several blood samples were taken from 25 to 48 h after calving, in which case the sample obtained closest to 36 h after calving was used. The blood sample representing 4 DIM was collected at 96 h in 4 studies but could be between 73 and 96 h after calving in other studies. Correlations between blood Ca concentration nadir and blood Ca concentrations at 2 and 4 DIM were also determined. Not all studies had collected blood samples at both 2 and 4 DIM.

The risk of subclinical hypocalcemia (SCH) at 2 and 4 DIM was defined as blood Ca concentration < 2.00 mM. Dyscalcemia was defined as blood Ca concentration < 2.20 mM at 4 DIM and is associated with increased risk of culling in dairy cows (4). The incidence of dyscalcemia was determined for the cows in those studies providing blood Ca concentrations at 4 DIM. These data were analyzed by logistic regression using generalized linear mixed-effects models with the GLIMMIX procedure of SAS (SAS 9.4) fitting a binary distribution. The models included the fixed effects of urine pH category (≤ 5.75, 5.76 to 6.25, 6.26 to 6.75, 6.76 to 7.25, 7.26 to 7.75, and ≥ 7.76), parity group (2 vs. 3 vs. ≥ 4), and the interaction between urine pH category and parity group, and the random effect of experimental study. The Kenward-Roger method was used to calculate the approximate denominator degrees of freedom for the *F* test in the statistical models. The predicted probabilities (LSM and respective SEM) in the generalized linear mixed-effects models were obtained with the ILINK option in SAS. The Tukey–Kramer method was used for adjustment for multiple comparisons. Values for blood Ca concentrations presented are the least square means ± standard errors.

## 3 Results

### 3.1 Time after calving when Ca nadir was observed in the study cows

In 495 of the 660 cows (75%), the Ca nadir occurred during the first 24 h after calving. The Ca nadir occurred in 119 cows (18%) between 25 and 48 h after calving, in 25 cows (3.8%) between 49 and 72 h after calving, in 9 cows (1.4%) between 73 and 96 h after calving and in 12 cows (1.8%) after 96 h. Forty-one cows (6.2% of the 660 total cows) had blood Ca concentration ≤ 1.25 mM, which can be indicative of a cow with clinical hypocalcemia or milk fever (1). Seventeen cows had blood Ca nadir below 1.00 mM. Table 2 presents, within each of the 9 studies that contributed prepartum urine pH and post-partum blood Ca concentrations, the parity distribution, number of cows diagnosed with clinical hypocalcemia within each parity, milking frequency/day, and the frequency of urine pH determination per week.

### 3.2 Effect of prepartum urine pH and parity on Ca nadir and sub-clinical hypocalcemia incidence

Across all 660 cows, urine pH category and parity had significant effects (*p* < 0.001) on Ca nadir and the percentage of cows with Ca nadir below 2.00 mM which categorized them as having SCH (Table 3). The mean Ca nadir of cows in the prepartum urine pH ≥ 7.76 category was 1.66 ± 0.03 mM, which was significantly lower (*p* < 0.05) than in cows in the more acidic urine pH categories. The Ca nadir of cows in urine pH categories more acidic than 7.75 were statistically similar (*p* > 0.05). As parity of the cows increased the blood Ca concentration nadir of the cows decreased significantly (*p* < 0.05), being 1.99 ± 0.03 mM for 2nd parity cows, 1.87 mM ± 0.04 for 3rd parity cows, and 1.70 ± 0.04 mM for ≥ 4th parity cows (Table 3).

The percentage of cows with Ca nadir below 2.00 mM, indicative of SCH, was highest in cows with prepartum urine pH above 7.76 (*p* < 0.05). Cows with prepartum urine pH below 7.75 had a statistically similar incidence of SCH (*p* > 0.05). As parity increased the percentage of cows with Ca nadir below 2.00 mM increased significantly (*p* < 0.05), being 41 ± 6% for 2nd parity cows, 63 ± 6% for 3rd parity cows and 87 ± 4% for ≥ 4th parity cows (Table 3).

### 3.3 Associations between prepartum urine pH, parity, and Ca nadir, and blood Ca concentrations observed at 2 and 4 DIM

Blood Ca concentration at 2 DIM was reported for 570 cows. The percentage of cows with SCH at 2 DIM was highest (43 ± 4%) in those cows with prepartum urine pH ≥ 7.76. Incidence of SCH at 2 DIM of cows with prepartum urine pH between 7.25 and 7.75 was 29 ± 7%, and SCH incidence in cows in the prepartum urine pH 5.76 to 6.25 category was 31 ± 5%, and did not differ from that of cows in the prepartum urine pH ≥ 7.76 category (*p* > 0.05). Cows in the other urine pH categories (between 6.76 and 7.25, between 6.26 and 6.75, and < 5.75) had significantly lower incidences of SCH at 2 DIM (*p* < 0.05) (Table 3). It is important to note that SCH at 2 DIM was statistically similar in all cows with urine pH below 7.75 (*p* > 0.05) (Table 3).

As parity increased the percentage of cows with SCH at 2 DIM increased significantly only for the cows in their ≥ 4th parity, being 20 ± 3% for 2nd parity cows, 27 ± 4% for 3rd parity cows and 45 ± 4% for ≥ 4th parity cows (*p* < 0.05).

Across the 534 cows with recorded blood samples at 4 DIM, the incidence of dyscalcemia at 4 DIM was not statistically different across the urine pH categories (*p* > 0.05). Cows in their 2nd parity exhibited less dyscalcemia than cows in their 3rd or ≥ 4th parity (*p* < 0.05) (Table 3).

There was essentially no association of urine pH with blood Ca concentrations at 2 DIM or at 4 DIM (Pearson coefficient of correlation values below −0.10).

### 3.4 Blood Ca nadir (mM) as affected by interaction of parity and urine pH category

Table 4 depicts blood Ca nadir concentrations for the subsets of 298 2nd parity cows, 195 3rd parity cows, and 167 ≥ 4th parity cows as affected by urine pH category and parity. As parity increased, the LS mean blood Ca concentration nadir within each urine pH category decreased, The lowest mean nadir blood Ca concentrations across all parity groups were seen when prepartum urine pH was ≥ 7.76 (*p* < 0.01). The mean blood Ca concentration nadir was significantly greater in 2nd and 3rd parity cows in the cows with prepartum urine pH between 7.26 and 7.75 (*p* < 0.05). Reducing urine pH further did not cause a statistically significant increase in blood Ca concentration nadir in 2nd and 3rd parity cows (*p* > 0.05).

**Table 4:** LS mean ± standard error blood Ca concentration nadir (mM) as affected by parity and urine pH category.

Urine pH category	2nd parity *N* = 298	3rd parity *N* = 195	≥ 4th parity *N* = 167
≥ 7.76	1.84 ± 0.04^b^ *N* = 90	1.67 ± 0.05^b^ *N* = 58	1.48 ± 0.05^c^ *N* = 53
7.26 to 7.75	1.98 ± 0.06^a^ *N* = 25	1.91 ± 0.08^a^ *N* = 14	1.65 ± 0.07^b^ *N* = 19
6.76 to 7.25	2.04 ± 0.07^a^ *N* = 20	1.84 ± 0.08^a^ *N* = 15	1.76 ± 0.08^ab^ *N* = 14
6.26 to 6.75	2.02 ± 0.05^a^ *N* = 29	1.95 ± 0.08^a^ *N* = 16	1.84 ± 0.07^a^ *N* = 22
5.76 to 6.25	2.04 ± 0.05^a^ *N* = 45	1.89 ± 0.06^a^ *N* = 34	1.79 ± 0.06^ab^ *N* = 26
≤ 5.75	2.03 ± 0.04^a^ *N* = 89	1.94 ± 0.05^a^ *N* = 58	1.66 ± 0.06^b^ *N* = 33

*N* = number of cows in each table cell. ^a, b, c^Within parity column, different superscripts denote differences in the continuous LS Means of blood Ca concentration nadir across the urine pH categories after correction by the method of Tukey–Kramer for multiple comparisons (*p* < 0.05).

Among ≥ 4th parity cows, those cows in the urine pH categories more acidic than 7.75 had significantly increased Ca nadir when compared to cows with urine pH above 7.75 (*p* < 0.05). Cows in their ≥ 4th parity with urine pH between 6.26 and 6.75 had significantly greater Ca nadir than did cows with urine pH between 7.26 and 7.75 or cows with urine pH below 5.75 (*p* < 0.05). However, Ca nadir was not different in ≥ 4th parity cows in prepartum urine pH categories 5.76–6.25, 6.26–6.75, and 6.76–7.25 (*p* > 0.05) (Table 3).

**Figure 1:**
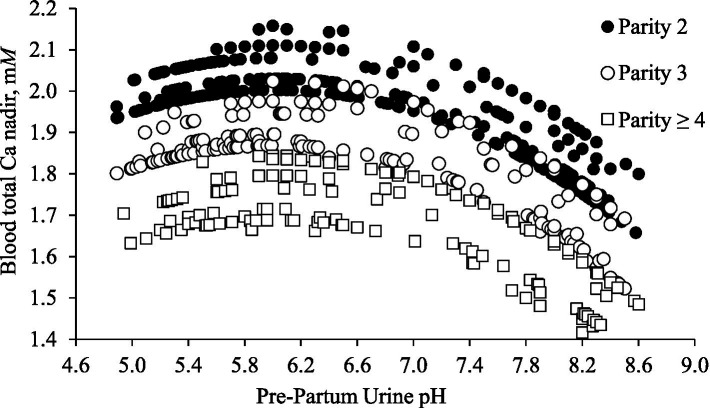
The mixed model predicted nadir concentration of total Ca in blood postpartum according to urine pH prepartum and parity group of 660 cows from 9 experimental studies. The mixed model included the fixed effects of urine pH linear (*p* < 0.001), urine pH quadratic (*p* < 0.001) and parity group (*p* < 0.001) and the random effect of experimental study.

The mixed model predicted nadir concentration of total Ca in blood postpartum according to urine pH prepartum and parity group of 660 cows from 9 experimental studies is depicted in Figure 1. The mixed model, which included the random effect of experimental study, determined the fixed effects of urine pH linear (*p* < 0.001), urine pH quadratic (*p* < 0.001) and parity group (*p* < 0.001) were significantly associated with Ca nadir. There was a quadratic association between prepartum urine pH and blood Ca concentration nadir.

### 3.5 Correlations between blood Ca concentrations at nadir and at 2 and 4 DIM

**Figure 2:**
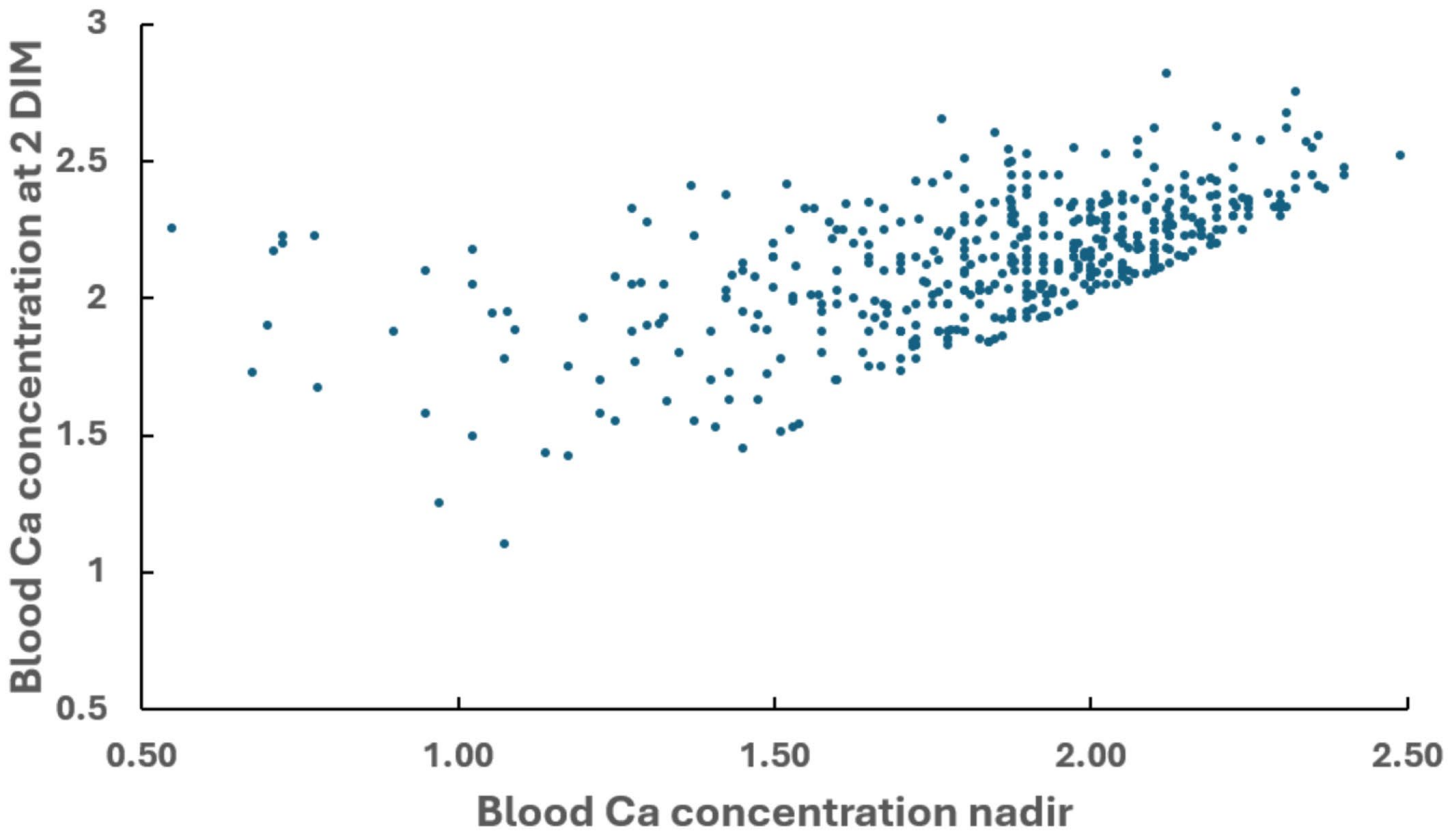
Correlation between blood Ca concentration nadir observed after parturition and blood Ca concentration observed at 2 DIM in multiparous cows. Pearson coefficient of correlation = +0.589.

Blood Ca concentration at 2 DIM was provided for 570 cows. The correlation between the blood Ca nadir of 463 cows and the blood Ca concentration at 2 DIM is presented in Figure 2. We removed 107 of the 570 cows from this correlation analysis because blood Ca concentration at 2 DIM was also the Ca nadir. The correlation coefficient was positive and relatively strong (r = + 0.59). Mean blood Ca concentration nadir in this 463-cow data set was 1.83 mM, and by 2 DIM the mean blood Ca concentration increased to 2.14 mM.

**Figure 3:**
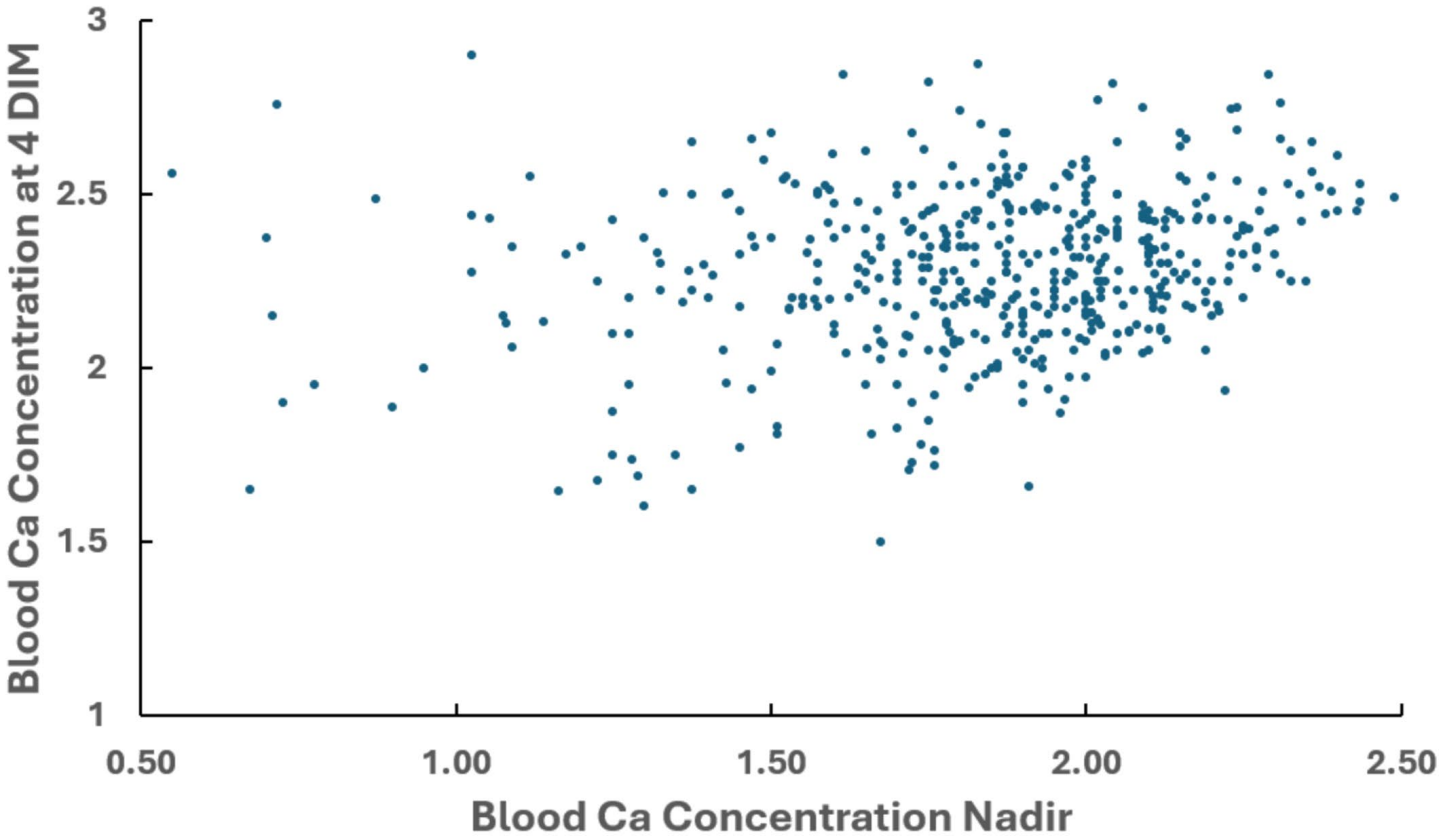
Correlation between blood Ca concentration nadir observed after parturition and blood Ca concentration observed at 4 DIM in multiparous cows. Pearson coefficient of correlation = +0.30.

Blood Ca concentration at 4 DIM was available from 534 cows. The correlation between the blood Ca nadir concentration and the blood Ca concentration at 4 DIM for 522 cows was significant (r = + 0.30) (Figure 3), but weaker than between Ca nadir and blood Ca concentration at 2 DIM. Twelve cows were removed from this analysis because blood Ca concentration at 4 DIM was also the Ca nadir. The mean blood Ca nadir concentration of cows in this 522-cow data set was 1.82 mM and the mean blood Ca concentration at 4 DIM was 2.29 mM.

**Figure 4:**
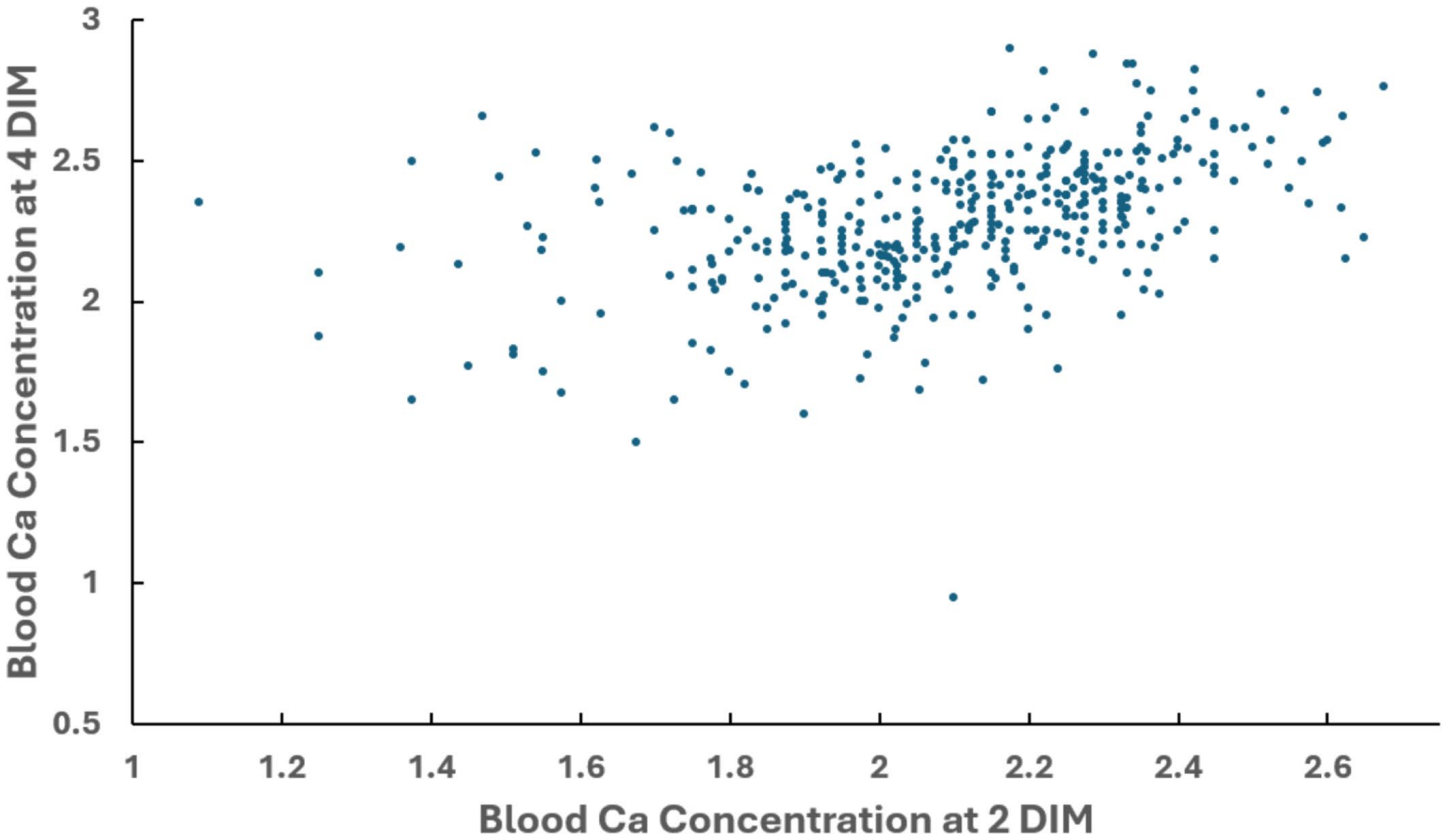
Correlation between blood Ca concentration observed at 2 DIM and blood Ca concentration observed at 4 DIM in multiparous cows. Pearson coefficient of correlation = +0.428.

The blood Ca concentration at 2 DIM and the blood Ca concentration at 4 DIM was available for 461 cows. The correlation between blood Ca concentration at 2 DIM and blood Ca concentration at 4 DIM was r = + 0.43, with mean blood Ca concentration at 2 DIM of 2.09 mM and at 4 DIM of 2.26 mM (Figure 4).

## 4 Discussion

Increasing parity of the cow had a strong negative effect on Ca nadir and the incidence of subclinical hypocalcemia and dyscalcemia in the cows, confirming studies demonstrating greater risk of hypocalcemia as cows age (27). We suggest that future publications examining the effects of diet or other interventions on periparturient blood Ca concentrations be sure to allocate cows to treatments matched by parity, since this has a large effect on blood Ca concentrations.

Lopera et al. (28) determined that urine pH below 6 was associated with significant reductions in DMI in cows. As DCAD decreases and urine pH approaches the physiologic limit of the kidney to excrete acid, there is a reduction of DMI to allow the cow to compensate for the diet anion excess and avoid entering a state of uncompensated metabolic acidosis (17). During metabolic acidosis the respiratory rate can increase CO_2_ loss to help control blood pH.

Cows with urine pH below 5.9 typically have a large increase in urine ammonium concentration. The renal tubule cells deaminate the amino acid glutamine and secrete the ammonia into the urine which neutralizes hydrogen ions as it is converted to ammonium. This allows the kidney to excrete more hydrogen ions without reducing urine pH further, which might damage the renal tubular epithelium (17).

Reducing urine pH below 7, with consequent renal tubular acidosis, reduces the ability of the kidneys to reabsorb Ca from the glomerular filtrate, thus resulting in increased urinary Ca loss as urine pH becomes more acidic (1, 17, 29). Higher urinary Ca loss with increasing acidity of the urine becomes especially evident if urine Ca/creatinine concentrations are determined to account for concentration of the urine (29). This is one of the many mechanisms for increased Ca flux in the body when cows are fed prepartum acidogenic diets that is suggested to prevent hypocalcemia postpartum. Thus, one expects that a more highly acidic urine pH prepartum should improve blood Ca postpartum. However, analyses of the 660-cow data set does not support the concept that lowering urine pH below 6, when urine Ca loss is reported to be greatest (29, 30), results in increased blood Ca nadir over that of cows with urine pH above 6.25, especially in ≥ 4th parity cows.

Parity was a greater influence on Ca nadir and SCH than urine pH. There was considerable variation in blood Ca concentrations within each urine pH category examined. The different methods used to determine blood Ca concentration and urine pH likely contribute to the variability in our data. Studies differed in the frequency of blood sampling after calving, which reduces the precision of identifying the blood Ca nadir. The time prior to parturition when the urine pH was evaluated varied across studies. The urine specific gravity (dilute or concentrated urine) was not considered in these studies, which also influences urine pH. Care was not always taken to prevent carbon dioxide from escaping the urine sample prior to pH determination (31). As Leno et al. (29) demonstrate, the creatinine concentration in the urine can greatly increase the value of urine calcium excretion evaluation and would likely improve hydrogen ion secretion evaluation in a similar fashion. More precise methods of urine hydrogen ion content would, unfortunately, detract from the field utility of this test.

Another limitation of urine pH testing, particularly when values are very high (above 8.2) or very low (below 5.9), is that it is less likely to reflect the blood pH and the pH of the extracellular fluid bathing parathyroid hormone sensitive tissues such as bone and kidney. The kidney, along with the respiratory system, functions to maintain blood pH as close to normal as possible. Blood pH is normally 7.40 to 7.45 in cows that are not fed anionic salts. At pH 7.40, the hydrogen ion concentration of the blood is 4 × 10^−8^ M (32). Blood pH below 7.15 or above 7.55 is generally not compatible with life. This equates to hydrogen ion concentrations in blood above 7 × 10^−8^ M (severe acidosis) and below 2.8 × 10^−8^ M (severe alkalosis). No other element is as tightly controlled as the hydrogen ion concentration of blood. The kidney has a limit to the amount of acid (or alkali) it can excrete. Once that limit is reached, the blood pH could continue to decrease (or rise) further, without altering urine pH. When the kidney has reached the limit of its ability to compensate for acidosis, the blood pH decreases rapidly, placing the cow in a state of uncompensated metabolic acidosis. Gelfert et al. (33) demonstrated that treatment of cows with increasing amounts of anionic salts to simulate diets with up to – 432 mEq/kg DM resulted in blood pH values indicative of severe systemic acidosis (average blood pH was 7.24). However, urine pH values remained above 5.8. Constable et al. (17), Gelfert et al. (33), and Gelfert et al. (34) demonstrated that determination of urine net acid (or base) excretion and ammonium excretion more precisely indicate changes in blood pH and blood base excess than does urine pH. The presence of more than 20 mEq/L of ammonium in urine is also suggestive of a cow that is highly acidotic (17). Unfortunately, the methods used to measure net acid excretion or urine ammonium concentrations are not readily applicable on farms. Though urine pH has a limited value as an index of blood pH, especially at the extreme margins of urine pH, at present it is the most practical measure of acid/base status for on-farm evaluation of the anionic diet.

We conclude that the hypothesis that periparturient blood Ca concentration was linearly inversely related to prepartum urine pH must be rejected. The response is curvilinear, and the lowest urine pH values are not associated with the highest periparturient blood Ca concentrations.

The nutritionist and veterinarian should be aware that achieving the optimal urine pH of 6.25–6.75 for older cows will be difficult as small changes in dry matter intake will cause variation among the cows. However, the data presented here suggest that significant decreases in blood Ca nadir will occur only if urine pH falls below 5.75 or rises above 7.25 in the older cows (> 4th parity).

These data support many earlier observations that hypocalcemia on the first day of lactation is common. McArt and Neves (4) refined the definitions of hypocalcemia in early lactation. They observed many cows with blood Ca concentration below 2.0 mM at 1 DIM had increased blood Ca concentration above 2.0 mM by 2 DIM; they had “transient hypocalcemia” and were the highest milk producers in that study. However, cows that remained subclinically hypocalcemic at 2 DIM had an increased risk of becoming “persistently hypocalcemic” through the first 4 DIM. McArt and Neves (4) also demonstrated that hypocalcemia at 4 DIM was a risk factor for culling. This implies that we should expect a high milk producing cow to have some degree of hypocalcemia on the first d of lactation. However, severe hypocalcemia, as observed in cows with milk fever, should be avoided as these cows also have more prolonged hypocalcemia and great risk of secondary metabolic and infectious disease (2, 5). Although the overall correlation between blood Ca concentration at 2 DIM and the blood Ca concentration at 4 DIM was positive indicating that for most cows their blood Ca concentration improved during that period, 56 cows experienced a blood Ca decrease of more than 0.10 mM from 2 to 4 DIM. The average blood Ca decrease of these 56 cows was 0.26 mM.

Dyscalcemia, defined as blood total Ca < 2.20 mM at 4 DIM, is associated with decreased pregnancy at first AI and pregnancy rate to 150 DIM (35) and with decreased postpartum activity and rumination (36). In our subset of 534 cows with Ca measured at 4 DIM, 35% were dyscalcemic (Table 2). The prevalence of dyscalcemia was lower in cows entering their second parity than in older cows. Of the 56 cows that exhibited a blood Ca decrease of ≥ 0.10 mM between 2 and 4 DIM, 42 (75%) were dyscalcemic. The correlation between Ca nadir and blood Ca concentration at day 4 was + 0.30, which is not considered strong. Factors beyond the blood Ca concentration nadir or blood Ca concentration at 2 DIM, such as parity or inflammation, also influence blood Ca concentration at 4 DIM (37, 38). Unfortunately, discerning what those factors are that cause blood Ca concentration to decline between 2 and 4 DIM cannot be determined from the compiled data and will have to be the subject of future research.

There was essentially no association of urine pH with blood Ca concentrations at 2 DIM or at 4 DIM (Pearson coefficient of correlation values below −0.10). In addition to the problems associated with urine pH determination as an index of blood pH described above, another problem confounding analysis of these associations resides in variation of the time label for blood samples among the studies. In the present studies, a 1 DIM blood sample was obtained in the first 24 h after calving and for 73% of the cows studied, this sample had the lowest blood Ca concentration measured. Several studies had more than one blood sample during the initial 24 h after calving, providing a more precise estimate of the blood Ca concentration nadir for each cow. Importantly, in several of the studies, a sample identified as a 2 DIM sample, could have been obtained any time between 25 and 48 h after calving. However, the blood Ca concentration in a sample obtained at 25 h after calving is likely to be considerably lower than in a sample obtained 47 h after calving (22, 28, 29, 39).

## 5 Conclusion

On farm determination of urine pH should continue to be encouraged as a means of assessing the adequacy of anion supplementation to the prepartum diet. The largest gain in the concentration of Ca in the blood of the newly calved cow will be made by simply reducing the degree of alkalinity of the urine as the degree of hypocalcemia experienced is significantly increased for most cows when urine pH is above 7.75. Reducing urine pH below 6.25 offers no greater benefit to periparturient blood Ca concentration in any multiparous cows. Older cows, ≥ 4th parity, may require a higher addition of anions to the diet to reduce urine pH below 7.25 for the greatest improvement of periparturient blood Ca concentration. In these older cows, urine pH below 5.75 had a detrimental effect on blood Ca Nadir.

## Data Availability

The data analyzed in this study is subject to the following licenses/restrictions: the data may be found in each of the previously published studies cited in Table 1. Requests to access these datasets should be directed to JG, jpgoff55@gmail.com.
